# Using Molecular Mechanics to Predict Bulk Material Properties of Fibronectin Fibers

**DOI:** 10.1371/journal.pcbi.1002845

**Published:** 2012-12-27

**Authors:** Mark J. Bradshaw, Man C. Cheung, Daniel J. Ehrlich, Michael L. Smith

**Affiliations:** 1 Department of Mechanical Engineering, Boston University, Boston, Massachusetts, United States of America; 2 Department of Biomedical Engineering, Boston University, Boston, Massachusetts, United States of America; Johns Hopkins University, United States of America

## Abstract

The structural proteins of the extracellular matrix (ECM) form fibers with finely tuned mechanical properties matched to the time scales of cell traction forces. Several proteins such as fibronectin (Fn) and fibrin undergo molecular conformational changes that extend the proteins and are believed to be a major contributor to the extensibility of bulk fibers. The dynamics of these conformational changes have been thoroughly explored since the advent of single molecule force spectroscopy and molecular dynamics simulations but remarkably, these data have not been rigorously applied to the understanding of the time dependent mechanics of bulk ECM fibers. Using measurements of protein density within fibers, we have examined the influence of dynamic molecular conformational changes and the intermolecular arrangement of Fn within fibers on the bulk mechanical properties of Fn fibers. Fibers were simulated as molecular strands with architectures that promote either equal or disparate molecular loading under conditions of constant extension rate. Measurements of protein concentration within micron scale fibers using deep ultraviolet transmission microscopy allowed the simulations to be scaled appropriately for comparison to *in vitro* measurements of fiber mechanics as well as providing estimates of fiber porosity and water content, suggesting Fn fibers are approximately 75% solute. Comparing the properties predicted by single molecule measurements to *in vitro* measurements of Fn fibers showed that domain unfolding is sufficient to predict the high extensibility and nonlinear stiffness of Fn fibers with surprising accuracy, with disparately loaded fibers providing the best fit to experiment. This work shows the promise of this microstructural modeling approach for understanding Fn fiber properties, which is generally applicable to other ECM fibers, and could be further expanded to tissue scale by incorporating these simulated fibers into three dimensional network models.

## Author Summary

There is growing awareness of the role of mechanical properties within biological tissues. Cells both generate force and are sensitive to applied forces, however nuanced sensitivity to externally applied forces also extends outside the cell to the fibrous structural proteins of the extracellular matrix. It has been shown that stretching these proteins under force can change their biochemical properties in a way that impacts tissue function. In this work we were able, for the first time, to measure the concentration of protein within fibronectin extracellular matrix fibers. This key measurement then enabled us to evaluate a model that links mechanical properties of fibers directly to molecular structural changes that form the physical basis for force sensitivity. The model was found to be predictive of fiber mechanical properties without fitting. This combination of modeling and experiment also offers insights into molecular forces, as well as estimates of fiber hydration and porosity.

## Introduction

Natural fibrillar materials possess remarkable mechanical properties that are tightly tuned to their functions, and great strides have been made toward achieving a quantitative understanding of the mechanics of these biological filaments. Detailed studies have shown how mechanical forces affect single molecules [1], [2], single fibers [3], [4], and fiber networks [5], [6] in a wide range of biological materials [7], [8], [9], [10], [11], but it is difficult in many systems to link molecular properties to fiber behavior. Full atomistic simulation in the same length and time scales found in the natural environment of these materials requires computational power that is not currently available. One solution is to address these challenges with microstructural models that link molecular and fiber length scales through comparison to experimental data. Several excellent examples of this approach have been recently published (e.g., [8], [11]), but it is important to note that modeling concepts are often specific to the material of interest and overall objective of this type of comparison. Our goal in this study was to use a microstructural model to determine how dynamic molecular conformational changes and the intermolecular arrangement of molecules within a fibrous material affect the bulk mechanical properties of fibronectin (Fn) fibers found in the extracellular matrix (ECM) of living cells.

Fn fibers, which are present in the ECM during development, wound healing, and diseases such as cancer, would benefit from microstructural modeling that could provide insight into their intermolecular architecture. Fn has been characterized mechanically both at the single molecule level [1], [12], [13] and as isolated single fibers [4]. Fn fibers are mechanically loaded by cell contractile forces to the point of mechanical failure both *in vitro* [14], [15] and *in vivo* [16], and mechanical extension of Fn fibers substantially alters their biochemical properties [17], [18]. Thus, a comprehensive link between Fn molecular and fiber properties would provide mechanistic insight into the role of mechanical force in regulating exposure or deactivation of these binding sites, and hence provide insight into the mechanobiological functions of Fn matrix.

The Fn molecule is a large (∼500 kDa), dimeric, modular protein composed of folded domains arranged in a linear chain that are assembled by cells into fibers, a process that has been described in a series of excellent reviews [19], [20], [21]. The nature of the Fn/Fn crosslinking sites within fibers, as well as the molecular architecture of molecules within the fiber, remains poorly characterized. Measurements of the topography of Fn nanofibers by AFM suggest a regularly spaced architecture of molecules overlapping at the ends of the dimer arms [22]. Furthermore, electron microscopic imaging suggests that Fn fibers are composed of nanofibers only two molecules in cross-section, but which can be micrometers in length [23], [24], [25]. Several hypotheses explaining intermolecular organization have been put forth and at least 6 different Fn/Fn binding sites have been identified [19], [22], [26]. Unfortunately, studies of Fn/Fn binding sites using traditional approaches where molecules are adsorbed onto a surface (e.g., using a quartz crystal microbalance) or interact as freely diffusing molecules may not be predictive of intermolecular contact sites within Fn fibers. Thus, a major advantage of microstructural models applied to Fn fibers is the ability to interrogate how molecular arrangements affect bulk behavior, thus confirming that hierarchical structure is indeed important for this system as would be predicted from studies of other biological materials [5], [27].

Cell contractility generates forces up to 100 nN per focal adhesion [28], [29], and *in vivo* these forces are applied directly to the ECM. Fn fibers vary in diameter from 10 nm to µm in diameter. Although a single focal adhesion is sufficient to strain Fn nanofibers, numerous contacts with the matrix that may span multiple cells would be necessary to stretch the µm-sized fibers that are strained to mechanical failure [16], [30]. During the process in which Fn fibers are mechanically loaded by cell contractility, mechanical work is transferred into the molecular network in the form of changes in entropy and enthalpy as polymer chains are stretched, and as bonds are stretched or domains unfold and refold. The enthalpic changes are reflected in changes in both the quaternary and tertiary or even secondary structure of Fn, and substantial evidence indicates that these processes work in concert to produce the greater than 600% strains that are accommodated by Fn fibers prior to mechanical failure [30], [31], [32]. The challenge, however, is to interpret these conformational changes in the context of the super-molecular architecture of the Fn fiber. Thus, using probabilistic modeling with a foundation built on the mechanics of single molecules might be key to understanding super-molecular architecture.

Single molecule force spectroscopy has long relied on probabilistic Monte-Carlo models to describe conformationally derived extension of single proteins under force [33]. In this work we have applied a similar approach to the mechanics of molecular networks representing biological filaments under uniaxial stretch. Historically, a major limitation of this strategy has been the lack of a physical dimension that can be used to relate *in silico* with *in vitro* work. To overcome this limitation, we used novel measurements on the concentration of Fn within fibers to provide a physical dimension to our model, thereby permitting conversion of force and distance to stress and strain and providing direct comparison between *in vitro* and *in silico* stretching experiments. This approach provides insight into the conformational landscape that is traversed by Fn molecules throughout the full range of fiber strains and strongly suggests that Fn fibers are composed of a molecular architecture that leads to disparate mechanical loading during fiber strain. Remarkably, the model is capable of predicting both *in vitro* measured fiber forces and the molecular forces that are experienced by Fn molecules within strained fibers with a minimum of assumptions and no fitting parameters.

## Results

### UV absorption microscopy

**Figure 1:**
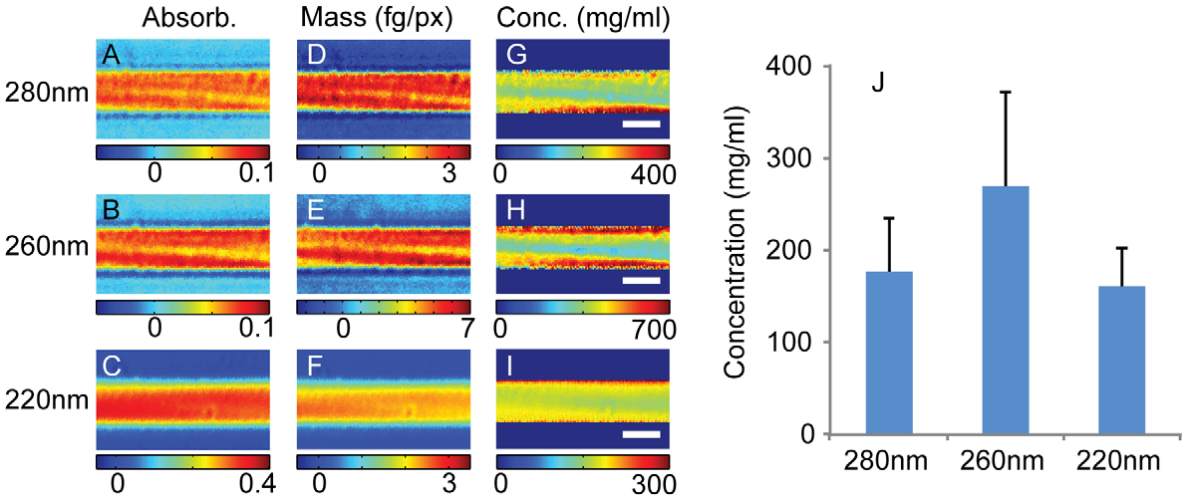
Deep ultraviolet transmission imaging of fibronectin fibers. Fn fibers (n = 8) were imaged in a deep ultraviolet transmission microscope at wavelengths 280 nm, 260 nm, and 220 nm. Panels A–C show optical density maps of the same Fn fiber at 280 nm (A), 260 nm (B), and 220 nm (C). These optical density maps were converted to maps of mass (D–F) using extinction coefficients calculated from the amino acid sequence of fibronectin (Uniprot P02751-9) and maps of concentration (G–I) by assuming the fibers have a cylindrical cross section to find the path length (scale bar 2 µm). Fn concentrations were averaged over a region in the center of the fiber to avoid edge effects due to errors in the assumed circular cross sectional profile. The resulting measurements of fibronectin concentration are shown in panel J, the error bars show one standard deviation. The Fn concentrations were 177±59 mg/ml, 270±102 mg/ml, and 160±42 mg/ml (mean ± SD) measured at 280 nm, 260 nm, and 220 nm respectively.

To provide a scale for the model, enabling comparison of *in silico* and *in vitro* data, we first sought to determine the concentration of Fn within single fibers. Despite decades of research into Fn biology, we are unaware of measurements on the concentration or water content of Fn fibers, although electron microscopic images of Fn indicate a dense structure [23], [26]. Thus, Fn fibers were imaged at high magnification using a deep ultraviolet transmission microscope at the wavelengths 280, 260, and 220 nm. The resulting optical density (Fig. 1A–C) was converted to protein concentration by assuming, as suggested by the mass maps, a circular fiber cross-section. The extinction coefficients were calculated from the amino acid sequence of the Fn protein for each wavelength using published values for the constituent amino acids [34], [35], [36] (704975 M^−1^cm^−1^, 412798 M^−1^cm^−1^, 4308680 M^−1^cm^−1^, for 280 nm, 260 nm and 220 nm respectively) and used to generate mass maps (Fig. 1D–F). The maps of Fn concentration (Fig. 1G–I) show some variation at the edges of the fibers, particularly at the two longer wavelengths where the optical density is on the order of 0.01 and small residual optical interference artifacts occur, but the optical density is relatively uniform in the center of the fiber. From a series of fibers (n = 8; with diameters 2.3 µm, 2.3 µm, 2.4 µm, 1.9 µm, 1.3 µm, 3.7 µm, 2.5 µm, 2.1 µm) all wavelengths agree fairly well with a concentration for the uniform central fiber core of 177±59 mg/ml, 270±102 mg/ml, and 160±42 mg/ml at 280 nm, 260 nm, and 220 nm (mean ± SD), respectively (Fig. 1J). However, due to the experimental verification of the extinction coefficient at 280 nm [37], we established the Fn density in these fibers as 177 mg/ml.

### Mechanical comparison of *in silico* and *in vitro* Fn fibers

Next, we sought to use these novel concentration measurements to provide a physical dimension to a microstructural model of a Fn fiber. Providing a physical dimension to our model fiber would allow a comparison of intrinsic material properties of *in silico* and *in vitro* fibers, for example by converting force versus displacement data to a plot of stress versus strain.

The design of a model is highly specific to the experimental objective, in this case to determine the effect of intermolecular architecture on Fn fiber properties. The Fn fiber model was formulated to incorporate the unfolding behavior of FnIII domains, which renders this model specific to materials with unfoldable domains. Each unfolding event adds significant length to the Fn molecule, 24% of the folded contour length of the entire dimeric molecule. The newly exposed region has dramatically different elastic characteristics due to the change in persistence length of the compact folded domain versus the unfolded strand. This newly formed spring is inserted in series with the original molecule and thus the stiffness of the pair is the harmonic mean of the folded and unfolded regions and will be dominated by the softer region. Domain unfolding is a rare event in FnIII domains due to the relatively large energetic barriers to unfolding (unfolding rate at zero force is of the order 1 per 100 sec [1]), meaning that these events must be treated as stochastic, far from equilibrium events.

In brief, *in silico* fibers consist of molecules modeled as worm like chains (Equation (1)) with starting contour lengths of 120 nm and persistence length of 14 nm that attach to adjacent molecules through freely-jointed nodes. The ability of Fn type III modules constituting the molecules to unfold was accounted for with the probability to unfold determined by Equation 2 and previously published single molecule data on Fn type III modules [1], [12], [38]. If an unfolding event occurred, a segment with a contour length of 32 nm and persistence length of 0.42 nm is inserted in series with the original molecule in which the contour length has been reduced by 3.5 nm. The fiber, with a starting length of 2.16 µm, was stretched in incremental steps of 0.67 nm, with a time step used for determining whether unfolding occurred of 0.73 ms at each increment. This provides an extension rate of 0.91 µm/sec, which is approximately similar to the previously published strain data of *in vitro* Fn fibers [4]. This model was then compared to previously published mechanical measurements made on a 3.0 µm diameter Fn fiber [4] in order to determine its predictive power.

As the *in vitro* stretching experiments used fibers that were µm in diameter, but the *in silico* stretching experiment used nm-scale fibers, we first had to scale the model data to match the number of Fn molecules measured with the ultraviolet transmission microscope. This scale factor was determined by assuming that *in vitro* Fn fibers were composed of a number of individual nanofibers. A second assumption was made about the end-to-end length of the Fn molecules within the nanofibers since assuming a longer or shorter starting end-to-end length of each molecule would have the net effect of increasing or decreasing, respectively, the total number of nanofibers constituting each fiber. The molecules were assumed to be extended to 90% of the initial contour length at 0% strain, which was chosen to provide consistency with both the previously published exposure of cryptic cysteines across the full range of Fn fiber stretch [31] and the ultimate stretch of the fibers before breakage.

**Figure 2:**
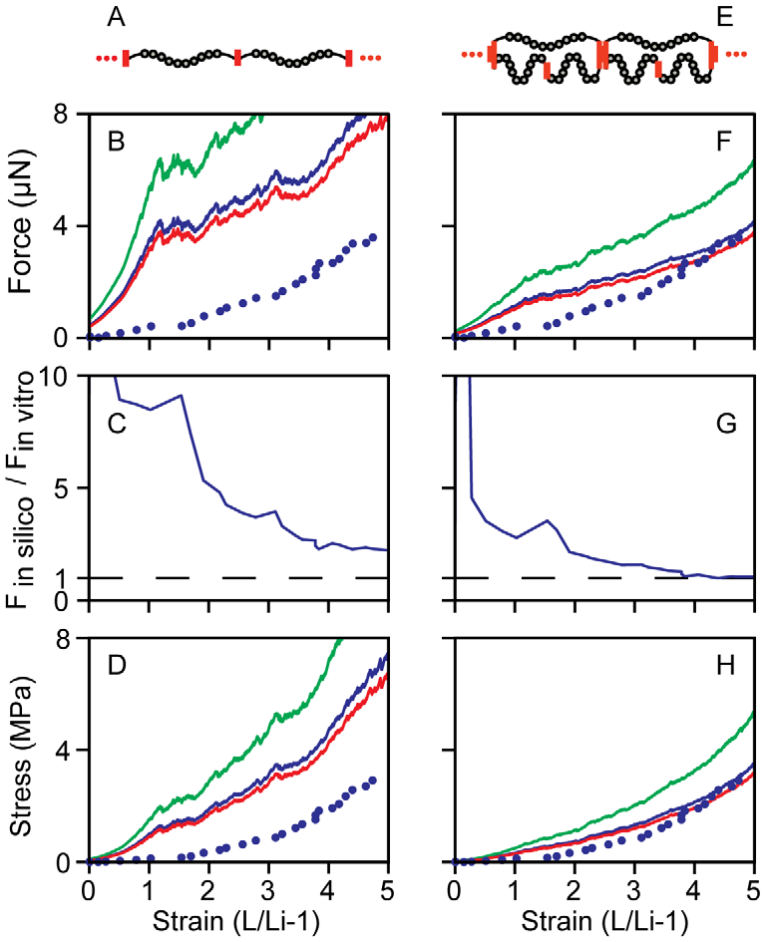
Simulated fibronectin fiber tension and stress compared to *in vitro* data. The tension of *in silico* fibers with 4 domains initially unfolded was scaled by the Fn concentration measured by UV microscopy. The model output is shown with experimental data from Klotzsch, 2009 [4]. The equal loading configuration (A) is shown scaled to the density determined by the UV microscope measurements (B) with wavelengths 280 nm (blue, 1.65e5 fibrils), 260 nm (green, 2.52e5 fibrils) and 220 nm (red, 1.49e5 fibrils), with the experimental data (blue dots). The tension along the equally loaded fiber (B) rises more quickly than the experiment before module unfolding begins to dominate the fiber mechanics slowing the rise in tension. Panel C shows the ratio of the model scaled by the 280 nm UV measurement to the experimental data. In the disparate loading case (E), the unequal distribution of forces causes the onset of unfolding at lower forces, smoothing the initial rise in tension (F). The ratio of force in the disparate simulation to the *in vitro* force reached was lower than in the equal fiber (G). The disparate loading case more closely matched the fiber tension of the experiment. The fiber stress was calculated for concentrations measured with each wavelength assuming a constant fiber volume and a fiber diameter of 3.0 µm at 0% strain (D, H). In the disparate loading condition (E) the force was scaled by the number of disparately loaded fibers (280 nm 5.50e4 fibrils; 260 nm 8.39e4 fibrils; 220 nm 4.98e4 fibrils).

Two molecular configurations were considered for *in silico* Fn fiber stretching. First, an equal loading configuration was considered where 20 molecules were joined end-to-end forming independent molecular strands (Fig. 2A). The force trace for the equal loading condition shows a steep initial rise before module unfolding starts to dominate the stretching behavior (Fig. 2B). Next, a direct comparison was made by plotting the ratio of *in silico* to *in vitro* forces, which drops progressively towards a value of 1 with increasing strain (Fig. 2C). Finally, the stress values in *in silico* and *in vitro* Fn fibers were compared in Fig. 2D by assuming a constant fiber volume during stretch, which is supported by data measuring reductions in fiber diameters during strain using fluorescence microscopy [4]. The second configuration considered was a disparate loading condition in which 20 segments containing one molecule in parallel with two molecules were connected end-to-end for a total of 60 molecules (Fig. 2E). The tension in the disparate loading condition has a smaller initial rise than that for equal loading as the load is initially transferred to the fraction of the total Fn molecules on the one molecule side of each 3-molecule structural unit (Fig. 2F). The ratio of *in silico* to *in vitro* forces also showed better correspondence for the disparate loading case (Fig. 2G) relative to the equal loading molecular architecture (Fig. 2C). Finally, the stress versus strain plot (Fig. 2H) indicates that the disparate loading condition is more predictive of *in vitro* fiber intrinsic stiffness than is the equal loading configuration. It is interesting to note in Fig. 2D that stress in the equal loading condition rose from 0 to ∼7 MPa, while the stress in the disparate loading fibers rose only from 0 to ∼4 MPa for the same concentration of Fn (Fig. 2H), indicating that molecular architecture can have a dramatic effect on the stress values achieved during material stretch. Finally, each of the simulations in Fig. 2 was performed by allowing 4 Fn type III domains to be already unfolded even in the fully relaxed state. We next studied the implications for the material properties of Fn fibers if unfolded domains are already present in fully relaxed fibers.

### Investigating the role of initially unfolded domains on fiber mechanics

**Figure 3:**
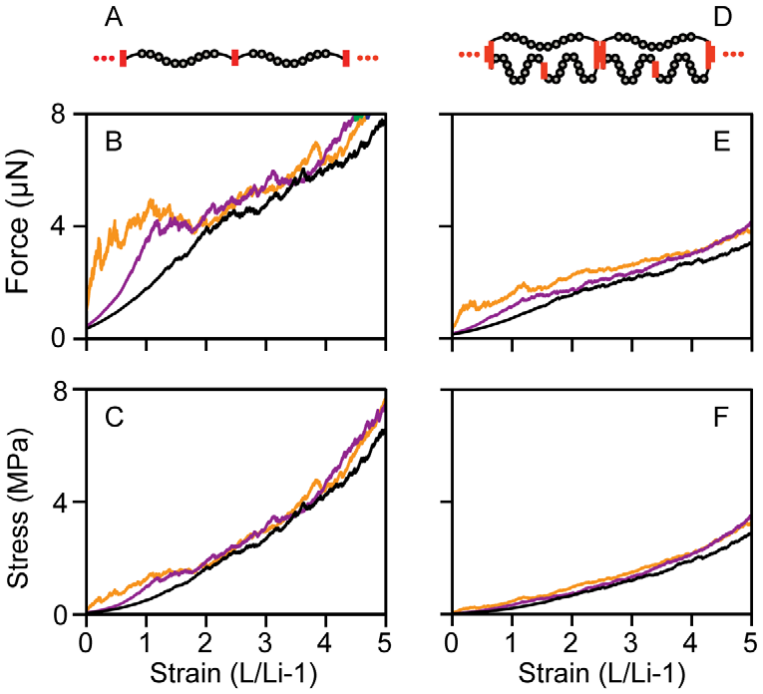
Comparison of fibers with initially unfolded domains. Fibers were simulated in two configurations, equal loading (A) and disparate loading (D), with either no domains initially unfolded (orange), 4 domains per molecule initially unfolded (purple) or 8 domains per molecule initially unfolded (black) for both the equally loaded fibers (B,C) and the disparate loaded fibers (E,F). Changes in the fiber Force (B, E) and Stress (C, F) were predominantly in the low strain region (<200% strain) where increasing the number of unfolded domains reduced the initial stiffness of the fiber.

A recent, thorough study of Fn domain unfolding in the ECM suggested that some domains may remain unfolded at all times within fibers [32]. Importantly, this suggests that some domains may be unfolded even in fully relaxed Fn fibers. One advantage of our comparison is that *in vitro* measured and *in silico* predicted Fn fiber stress values can be compared over the full range of Fn fiber strains. Importantly, the largest discrepancy between these two values occurred in the low strain regime. Thus, we sought to determine how having already unfolded FnIII domains altered the stress measurements in the low strain regime. The effect of these unfolded domains on the mechanical properties of fibers was investigated by simulating fibers with 0 domains, 4 domains (4×FnIII-12) and 8 domains initially unfolded (2×FnIII-2, 2×T-FnIII-3, 2×FnIII-9, 2×FnIII-12) (Fig. 3). The results of simulations with 0 and 8 modules initially unfolded compared to *in vitro* data are shown in Figure S1 and S2 respectively. The simulation was scaled to a 3.0 µm diameter using the measurement of Fn density at 280 nm. Increasing the number of initially unfolded domains made the fiber softer at low strains (<200%) while leaving the high strain behavior (>200%) unchanged, thus leading to a closer match between *in silico* and *in vitro* fiber force values. Comparisons at higher strains were unchanged regardless of the initial number of unfolded domains.

### Estimated single molecule forces in a fiber

**Figure 4:**
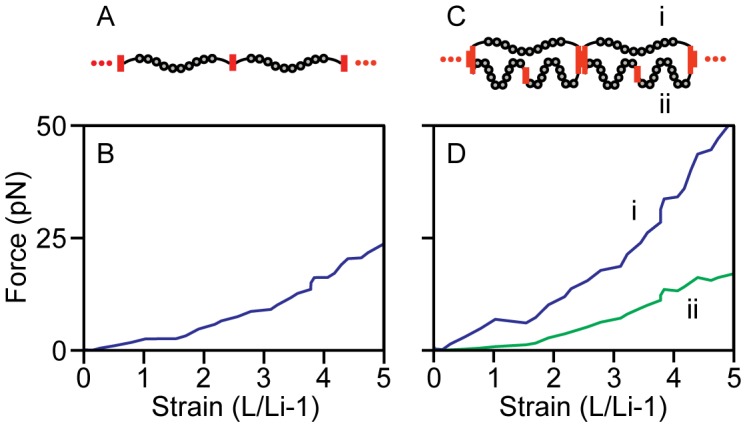
Prediction of molecular forces from *in vitro* measurements. The experimental results from Klotzsch 2009 were scaled using the UV absorption measurements of Fn concentration at 280 nm. The load on individual molecules in the equal loading configuration (A,B) was calculated by dividing the measured fiber tension by the calculated number of fibrils (1.65e5 fibrils). The loads in the disparate loading condition (C) were calculated by dividing the measured fiber tension by the number of disparately loaded fibrils (5.50e4 fibrils) then solving for the forces required to maintain the ratio between molecular forces found in the disparate loaded simulation (D). In the disparate loading condition there are two distinct populations of molecular forces. The more extended molecules (i) are under a higher load while the pair of molecules connected in series (ii) is shielded from force.

Finally, the microstructural model described here spans the molecular to fiber length scales and thus possesses the ability to estimate the molecular forces on individual molecules within the fiber. This is an important value due to its direct effect on Fn fiber structure, and until now the amount of force on Fn molecules within a fiber could only be estimated, for example by a comparison to the force of a single myosin motor [39]. Using the previous measurements, molecular forces in the equal loading configuration (Fig. 4A) rose from 0 pN at 0% strain to 22 pN at 473% strain using the concentration from the 280 nm UV measurements (Fig. 4B). Forces are identical between all molecules since they are arranged in series. In the disparate loading condition (Fig. 4C), forces in each 3-molecule structural unit differ between the one-molecule and two-molecule sides. The forces on the shorter, single molecule side of the segment (top molecule in Fig. 4C) rose from 0 pN at 0% strain to 47 pN at 473% strain while the tension in the two molecule side of the segment rose from 0 pN at 0% strain to 16 pN at 473% strain. Note that the total force in the nanofibers in the two configurations is different, consistent with the differences in the stress values of the two configurations at a given strain (Fig. 2D, H).

## Discussion

This study utilized a novel UV microscopic approach to measure the mass of Fn within single Fn fibers and then used this to refine a computational model to predict the bulk material properties of single Fn fibers under mechanical load. This microstructural model provided predictive power for a molecular architecture that results in disparate mechanical loading of molecules, but was less predictive for a model resulting in equal loading of the molecules. Furthermore, the estimate of Fn concentration in the fiber resulting from the measurements of mass provides a first estimate of the degree of packing found within Fn fibers and suggests that large molecules or aggregates would be excluded from the fiber. Finally, this multi-scale model is derived from a probabilistic framework defining the behavior of individual molecular domains as the origin of time dependence. This is a unique construction relevant in this case due to the molecular structure of Fn. Thus, the modeling approach used here may be used in the future to elaborate the mechanistic origins of viscoelastic properties of Fn fibers on longer, more physiologically relevant time scales.

Relatively simple models that span multiple scales can be very informative in explaining how molecular mechanics result in behavior at the macroscale. This has been shown for a wide variety of biological materials such as silk, fibrin, and nuclear lamina. The model developed here considered Fn fibers as perfect cylinders with repetitive molecular organization. However, defects are a common occurrence in nature, and should be considered in the context of ECM. Some insight into the effect of defects in Fn matrices can be understood from other fibrous network systems. For example, the nonlinear tension versus stretch behavior of fibrin fibers has been shown to strengthen fiber networks and prevent network failure by shielding small diameter fibers from excessive strain [6]. Similar multiscale structural motifs exist in the nuclear lamina [27] as well as spider silk where a series of molecular transitions give the fibers a highly nonlinear yielding then stiffening behavior [40]. This behavior has been demonstrated to make spider webs structurally more robust to damage from the environment or prey [5]. Fn matrices are analogous to these systems since Fn fibers show remarkable strain hardening properties [4]. Thus, smaller diameter fibers or fibers with imperfections should be shielded from high strains as the fiber hardens under load. In addition, the presence of defects in the molecular arrangement would create disparities in the loading of the Fn molecules. In light of this it is not a surprise that the mechanics of Fn fibers were better described by the disparately loaded case than the defect free equal loading case, and future work should address the presence of defects in Fn matrix.

The concentration measurements described here suggest that Fn molecules are tightly packed within Fn fibers. For a 3.0 µm diameter fiber at 0% strain, the concentration measured at 280 nm suggests that 1.65e5 molecules are present in cross-section, with 7.1 µm^2^ of cross-sectional surface area leading to 43 nm^2^ per molecule. Given that a molecule of Fn in cross section may occupy approximately 7 to 10 nm^2^ assuming a circle of 3 nm in diameter, this would suggest that approximately three-quarters of the Fn fiber is composed of solute. After extending a fiber with a starting diameter of 3 µm at 0% strain to a strain of 140%, the diameter would reduce to 1.9 µm, leading to a cross-sectional area of 18 nm^2^ per molecule. At higher extensions, a fraction of Fn type III domains would be unfolded giving them a substantially smaller cross-sectional surface area. This seems consistent with the relatively concentrated images of Fn fibers that have been qualitatively seen in electron microscope images of cell-derived matrix [23], [26]. The porosity of the fiber is currently not known, although this property is important to consider since Fn matrix is a reservoir for numerous growth factors [41]. The accessibility of the interior of Fn fibers to signaling molecules determines whether Fn matrix acts as a surface for adsorption or a sponge for absorption, and future work should attempt to determine the nm-scale accuracy of these µm-scale measurements. Whether Fn fibers are actually composed of bundled nanofibers remains to be determined, although small nanofibers that may only consist of 2 or 3 molecules in cross-section have been visualized with electron microscopic imaging [24], [25]. More intriguingly, immune-gold labeling of the EIIIA domain of cell-derived Fn suggested a qualitatively regular spacing of Fn molecules in Fn nanofibers [26], consistent with that used in our modeling approach here.

One unique feature of *in vitro* and *in silico* fiber stretching experiments is that comparisons can be made across the full spectrum of strain from relaxed to failure. Interestingly, we found that the early stages of fiber extension were substantially better modeled *in silico* when some FnIII domains were unfolded even in the fully relaxed state (Fig. 3). Although spontaneous unfolding is a relatively rare event for isolated FnIII domains in the absence of force [1], [42], [43], the presence of unfolded domains in presumably relaxed fibers is supported by a recent, extensive study of the folded state of FnIII domains within cell-made matrix [32]. At least two possible scenarios may contribute to the disrupted structure of FnIII domains in relaxed fibers. First, some FnIII domains could be unfolded due to their assembly into fibers, and this non-equilibrium conformation could be stabilized by the supermolecular architecture of the fiber. Second, it is possible that some of the nanofibers from which Fn fibers are composed remain in a state of prestress, even in the fully relaxed state. Since the data presented here strongly suggests that Fn molecules are disparately loaded during fiber stretch, having individual nanofibers that vary in their contour lengths but that possess similar end-to-end lengths would also result in disparate loading. This might lead to some nanofibers that are under mechanical stress and hence have some unfolded domains, but which cannot shorten due to the steric architecture and tight packing of the fiber. Ultimately, understanding the origin of these already unfolded molecules may give insight into the mechanism of Fn fiber assembly.

**Figure 5:**
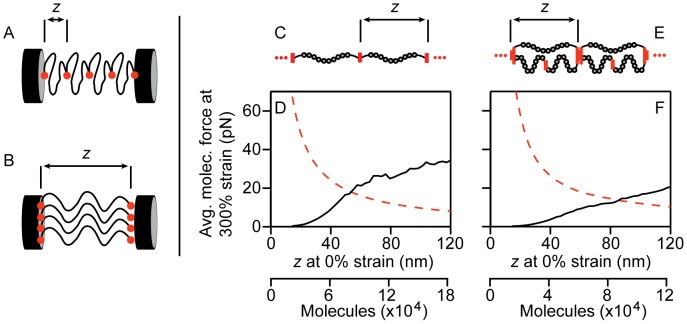
The influence of segment initial length assumption on the match between simulation and experiment. The assumption of initial molecular end-to-end length z impacts the calculation of the number of molecules in the cross section of the fiber. Small values of z (A) lead to fewer molecules in the fiber cross section than larger values of z (B) for the same Fn density. Thus, increasing values of z leads to a decrease in the average molecular force when scaling experimental whole fiber data to average molecular force (red dashed line in D and F). The value of z impacts the scaling of the simulated molecular strand by remapping the value of strain (black in D and F). The average molecular force at 300% strain estimated from scaling of experiment (red) and simulation (black) of equal loading configuration (C) and disparate loading configuration (E) is shown in plots (D) and (F) respectively. The value of z is directly correlated to the number of molecules in the fiber cross section. The equal loading configuration showed agreement at 54 nm of initial length. For the disparate loading configuration, the model matched experimental data at 86 nm initial length.

Our model *in silico* Fn fiber consists of a number of nanofibers that act together to provide the bulk properties of the fiber. Since the concentration measurement only provides a total number of molecules in the fiber, a starting end-to-end length had to be assumed for the molecules in each nanofiber in order to determine the total number of nanofibers in cross-section. For example, consider a simple Fn fiber composed of only 4 molecules. With a shorter starting end-to-end length, the fiber would be composed of a single nanofiber with 4 molecules in series (Fig. 5A). For a longer starting end-to-end length, the fiber would be composed of 4 nanofibers in cross section that are each only composed of a single molecule (Fig. 5B). Next, a comparison can be made between the force calculated using the WLC equation and nanofiber dimensions (the *in silico* prediction of nanofiber force) and the measurement of *in vitro* fiber properties that can be scaled down based on an assumption of the number of nanofibers in its cross section (the *in vitro* force). As a result, when the *in vitro*, experimental data is scaled to the force per molecule by dividing by the number of nanofibers in the cross section, the scaled down *in vitro* force per nanofiber predicts a dramatically large force for small starting end-to-end lengths but a very low force when end-to-end lengths are longer leading to more nanofibers in cross section (Fig. 5D, F, red dashed line). In stark contrast, the *in silico* prediction of nanofiber forces suggests very low forces for small starting end-to-end lengths that increases as end-to-end length is increased (Fig. 5D, F, black solid line). Thus, limiting the starting end-to-end length to permit extension only through loss of quaternary structure and not unfolding, as has been proposed [13], [44], gives low forces based on the *in silico* model, but would actually result in very large forces per nanofiber when the concentration measurements are scaled to the data of Klotzsch et al. [4]. Similar trends are seen whether the fiber is modeled as equally (Fig. 5C, D) or disparately loaded strands (Fig. 5E, F). Thus, this paradoxical result suggests that an intermediate starting end-to-end length leads to a best fit between the *in silico* predicted force and the force calculated from scaling the *in vitro* measured values of micron-sized fibers. Remarkably, the starting end-to-end length in the disparate case (72% of contour length) with the best match between *in silico* prediction and *in vitro* measurement was similar to the starting end-to-end length that we previously determined from cryptic cysteine exposure (90% of contour length), strongly supporting the hypothesis of disparate loading and an already partially extended conformation of a fraction of molecules in the fiber at 0% strain.

The predictive power of this *in silico* modeling approach is especially remarkable considering the limited number of assumptions used to generate the model. First, molecular refolding was not considered. Refolding of FnIII domains is a complex process that was not considered in our model since it leads to another alteration in contour length, effectively increasing the local force in that section of the fiber. However, we do not feel refolding would have an impact in the larger strain regime (i.e., greater than 300% strain) since molecular forces become excessive, thus prohibiting refolding [1], [43]. Steric interactions within the fiber were also not considered due to a lack of any knowledge about interactions between nanofibers. Our model only considered two possible molecular arrangements within the nanofiber, although disparate loading could occur with other molecular configurations. For example, fibers could be composed of nanofibers with identical end-to-end lengths between their connecting nodes, but different numbers of molecules and hence different contour lengths per nanofiber. Finally, our model did not consider interactions between adjacent nanofibers that would effectively cause additional viscous dissipation and higher forces during fiber stretch.

The combination of experiment and model described in this paper help elucidate the molecular origins of Fn fiber mechanics, demonstrating that a combination of domain unfolding and entropic stretching is sufficient to explain the extremely high extensibility of Fn fibers. The predictive capability of the model demonstrated relatively high accuracy over the available data set. Testing over an expanded data set which includes a wide range of strain rates and experimental conditions would validate domain unfolding as the source of time dependence in Fn fiber properties. A data set that contains fibers of widely varying diameters would allow us to identify behaviors that are emergent based on fiber scale. Also testing cell made Fn fibers would validate our *in vitro* fiber properties. Finally, this model could be translated to other polymer systems with force dependent conformational changes. The probabilistic approach used here might be more informative regarding time-dependent properties of fibers such as creep under constant load, and hence may be better suited to describe more physiologically relevant scenarios that synthetic and natural biomaterials experience *in vitro*.

## Methods

### UV microscopy

Fibronectin fibers were pulled from a drop of 1 mg/ml Fn solution in PBS using a previously developed technique [45], placed on quartz slide (Cat # CGQ-0640-01, Chemglass Life Sciences, Vineland, NJ) and mounted in PBS under a quartz coverslip. The UV imaging technique had been previously described and used to calculate protein and nucleic mass in adherent cell cultures [46]. In brief, the microscope is a Zeiss Axioskop that had been modified to accept a laser-induced plasma, high-pressure Xe gas light source (EQ-99, Energetiq Inc., Woburn, MA) via a fiber optic guide. The fiber output is directed through UV bandpass filters, (Omega Optical, Brattleboro, VT) with centers at 280, 265, and 220 nm (+/−5 nm) and then to a UV-Kond condenser (0.6 NA). Light is collected in a straight-through configuration by a 1.25 NA Ultrafluar 100X (Carl Zeiss AG), glycerin-immersion objective. Images of the fiber were captured by a UV-sensitive EMCCD camera (PhotonMax 512, Princeton Instruments, Trenton, NJ). This implies a diffraction-limited optical resolution of near 120 nm for the 1.2 NA Zeiss objective which, however, is degraded by about a factor of two due to inadequate oversampling at the CCD camera. The excellent fit to Beer's Law ultraviolet extinction has been well verified in Ref. [46] and is also apparent in the flat protein profiles seen for the cylindrical profile fits of Fig. 1 G–I. The method exposes the fiber sample to a low-dose-rate UV exposure [∼100 µW/(mm)^2^] that does not cause any apparent photochemical effects (in fact the method is successful for live-cell imaging over extended periods).

The absorption is related to concentration by Beer's Law. It has been shown that protein and nucleic acid dry masses can be decoupled using 220 nm and 260 nm absorption images [46]. UV absorption is wavelength dependent and is dominated by both protein and nucleic acid between 200 and 280 nm. A linear system of equation can be set up to solve for these two unknowns using two wavelengths. In the case of fibronectin fibers, the nucleic acid term can be dropped, allowing us to solve for protein concentration using the equation at each wavelength. The equation relating absorption to fibronectin concentration is
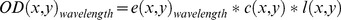
(1)where *OD* is the optical density, *ε* is the extinction coefficient for fibronectin, *c* is the concentration and the *l* is the thickness of the fiber at a given pixel (x,y). We used an extinction coefficient calculated using amino acid absorption for fibronectin at 280 nm [47] and 260 nm [34] and a coefficient calculated from a theoretical model of protein absorption for 220 nm [35]. The amino acid sequence used to calculate protein absorption is available at The Uniprot Consortium (http://www.uniprot.org; accession number P02751-9).

Images were acquired using WinVIEW software (Princeton Instruments) and analyzed off-line using custom-written, automated software algorithms in MatLab (Mathworks, Natick, MA). Images containing specimens and blank fields were obtained for each wavelength. Using automated software, absorption can be calculated by comparing the transmission of UV light through a blank field and a field with a specimen. After converting raw images into optical density maps, they were further processed to produce mass maps using the appropriate extinction coefficient. The dry mass of fibronectin fibers can be measured on a per-pixel basis. Concentration of fibronectin molecules was found by assuming the fibers were cylindrical with a diameter indicated by the fiber width in the image. The corresponding path length was given by 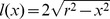 where *l* is the path length, *r* is the radius of the fibronectin fiber, and *x* is the orthogonal distance to the fiber axis.

### Mechanical model

We utilized a mechanical model of Fn fibers based purely on the entropic elasticity of polymer chains and the unfolding properties of FnIII domains that we described previously [31]. Briefly, the fiber was modeled as a network of molecules represented by worm-like chain (WLC) springs connected at nodes, with each spring obeying the WLC interpolation formula [48]

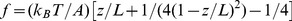
(2)with a persistence length *A* of 14 nm and an initial contour length *L* of 120 nm. In this formula *f* is the tension in the molecule, *z* is the molecular end-to-end length, *k_b_* is the Boltzmann constant, and *T* is temperature. Unfolding of FnIII domains was represented by a Bell's model unfolding probability [49], [50] according to
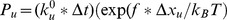
(3)where 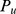 is the probability of unfolding, 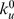 is the unfolding rate in the absence of force, 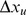 is the projected bond displacement at rupture and 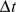 is the time interval. Parameters 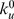 and 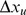 were determined by single molecule force spectroscopy of recombinant FnIII repeats [1] or estimated to match known unfolding forces (see [31] for a full list of values). In the event a FnIII domain unfolds, a length of 3.5 nm was subtracted from the contour length of that molecule and a length of 32 nm was added to another WLC with a persistence length of 0.42 nm, consistent with the persistence length of an amino acid chain [13] in series with the original molecule. Of the 15 unique FnIII domains we considered in our model Fn molecule, unfolding rate information exists for 9 of them. In order to compensate for unknown domains, the remaining missing domains were given the properties of FnIII12. The simulated stretch experiment was begun by holding the boundary nodes at a fixed length and increasing the boundary separation piecemeal at each time step to represent a constant extension rate. Importantly, we simulated a slow pulling rate of 0.91 µm/sec that is representative of the pulling rate used to stretch Fn fibers in vitro and which is more realistic than the extremely fast pulling rates required in atomistic models due to limited computational resources. At each time step the equilibrium network configuration was found by minimizing the strain energy of the network with respect to node positions. Then domain unfolding was tested by polling the unfolding probability of each folded FnIII domain with a random number to determine if an unfolding event occurred.

### Scaling model forces to fiber scale

The *in silico* Fn fiber stretching experiments only considered nanometer-scale fibers that were either 1 or 2 molecules in cross-section. However, the in vitro pulling experiments consisted of micrometer-scale fibers that contained an unknown number of nanofibers in cross-section. In order to compare these two, we used the molecular concentration measurements acquired with the deep ultraviolet transmission microscope to determine the number of nanofibers present within the in vitro fiber cross-section. In order to make this comparison, an assumption was made about the end-to-end length of the Fn molecules. Briefly, assuming a longer or shorter starting end-to-end length of each molecule would have the net effect of increasing or decreasing, respectively, the total number of nanofibers constituting each fiber. The molecules were assumed to be extended to 90% of the initial contour length at 0% strain, which was chosen to provide consistency with both the exposure of cryptic cysteines across the full range of Fn fiber stretch [31] and the ultimate stretch of the fibers before breakage. The concentration of Fn molecules from experiment was used to determine the number of molecules within the fiber cross-section. The two molecular configurations were scaled by different values to be consistent with the molecular concentration. The output of the equal loading configuration was scaled by the number of molecules in the fiber cross section. The disparate loading configuration in which one molecule was in parallel with two molecules was scaled by one third of the number of molecules in the fiber cross section. Scaling factors were calculated for the concentration measured with each of the three absorption wavelengths (Fig. 1J).

The forces applied to individual molecules within the experimental Fn fiber were estimated by scaling the experimental result from Klotzsch et al [4]. Two molecular configurations were considered, an equal loading scheme with independent single molecule strands and a disparate loading scheme where one molecule is joined in parallel with two molecules in series. Scaling in the equal loading configuration was accomplished by dividing in vitro fiber data by the number of molecules in a fiber cross section calculated above. For the disparate loading condition the experiment was scaled by dividing by the number of disparate loading fibrils calculated above and maintaining the ratio of force between the two populations of molecules in the disparately loaded model.

## Supporting Information

Figure S1 **Simulated fiber tension and stress with no domains initially unfolded compared to** ***in vitro*** **data.** The *in silico* data was scaled to a fiber diameter at 0% strain of 3.0 µm. The fiber was scaled according to measurements of fibronectin density made at 280 nm (blue), 260 nm (green), and 220 nm (red). Two fibril architectures were considered, an equal loading case (A) and a disparate loading case (E). The simulations were compared to *in vitro* measurements of fiber mechanics (blue circles) by Klotzsch *et al*. The tension in the equally loaded fiber (B) rose more quickly to a higher value than the disparate loading case (F). The difference between experiment and simulation was quantified by the ratio between force of the simulations (F*in silico*) and force of the experiment (F*in vitro*) for the equal loading case (C) and the disparate loading case (G). Stress was calculated for the simulation with the assumption of constant volume extension. Stress in the equally loaded fiber (D) was higher than the *in vitro* measurements over the strain range. Stress in the disparately loaded fiber (H) was lower than the equally loaded fiber and better approximated the *in vitro* data.
(TIF)

Figure S2 **Simulated fiber tension and stress with 8 domains initially unfolded compared to** ***in vitro*** **data.** The following 8 domains were initially unfolded (2×FnIII-2, 2×FnIII-12, 2×T-FnIII-3, 2×FnIII-9). The *in silico* data was scaled to a fiber diameter at 0% strain of 3.0 µm. The fiber was scaled according to measurements of fibronectin density made at 280 nm (blue), 260 nm (green), and 220 nm (red). Two fibril architectures were considered, an equal loading case (A) and a disparate loading case (E). The simulations were compared to in vitro measurements of fiber mechanics (blue circles) by Klotzsch *et al*. The tension in the equally loaded fiber (B) rose more quickly to a higher value than the disparate loading case (F). The difference between experiment and simulation was quantified by the ratio between force of the simulations (F*in silico*) and force of the experiment (F*in vitro*) for the equal loading case (C) and the disparate loading case (G). Stress was calculated for the simulation with the assumption of constant volume extension. Stress in the equally loaded fiber (D) was higher than the *in vitro* measurements over the strain range. Stress in the disparately loaded fiber (H) was lower than the equally loaded fiber and better approximated the *in vitro* data.
(TIF)
