# Supplementary figures and images for: Using Molecular Mechanics to Predict Bulk Material Properties of Fibronectin Fibers
Source: PLoS Comput Biol. 2012 Dec 27;8(12):e1002845. doi: 10.1371/journal.pcbi.1002845 (PMC3531316; doi:10.1371/journal.pcbi.1002845)

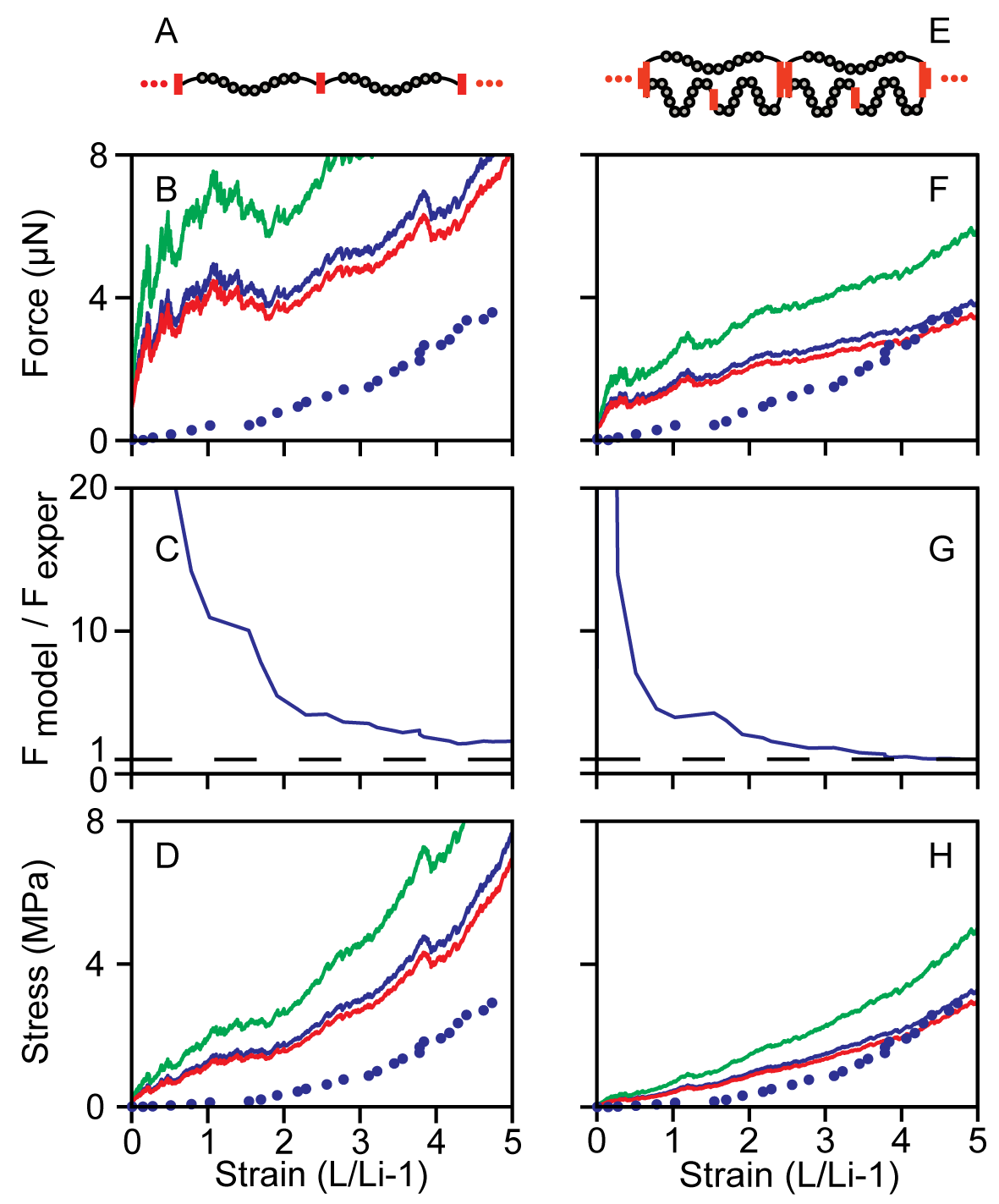

Supplement: Figure S1 — Simulated fiber tension and stress with no domains initially unfolded compared to in vitro data. The in silico data was scaled to a fiber diameter at 0% strain of 3.0 µm. The fiber was scaled according to measurements of fibronectin density made at 280 nm (blue), 260 nm (green), and 220 nm (red). Two fibril architectures were considered, an equal loading case (A) and a disparate loading case (E). The simulations were compared to in vitro measurements of fiber mechanics (blue circles) by Klotzsch et al. The tension in the equally loaded fiber (B) rose more quickly to a higher value than the disparate loading case (F). The difference between experiment and simulation was quantified by the ratio between force of the simulations (Fin silico) and force of the experiment (Fin vitro) for the equal loading case (C) and the disparate loading case (G). Stress was calculated for the simulation with the assumption of constant volume extension. Stress in the equally loaded fiber (D) was higher than the in vitro measurements over the strain range. Stress in the disparately loaded fiber (H) was lower than the equally loaded fiber and better approximated the in vitro data. (TIF) [file pcbi.1002845.s001.tif]

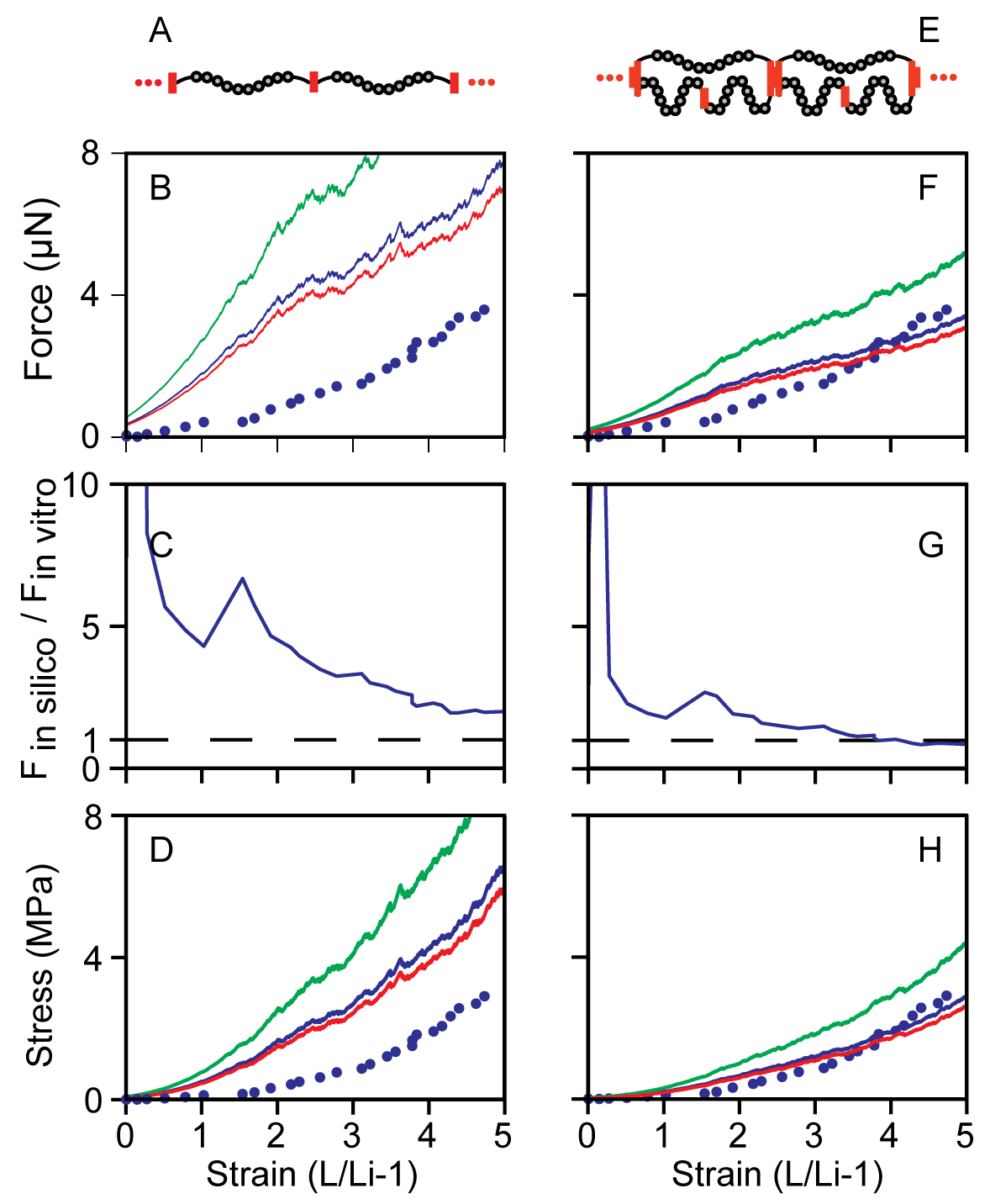

Supplement: Figure S2 — Simulated fiber tension and stress with 8 domains initially unfolded compared to in vitro data. The following 8 domains were initially unfolded (2×FnIII-2, 2×FnIII-12, 2×T-FnIII-3, 2×FnIII-9). The in silico data was scaled to a fiber diameter at 0% strain of 3.0 µm. The fiber was scaled according to measurements of fibronectin density made at 280 nm (blue), 260 nm (green), and 220 nm (red). Two fibril architectures were considered, an equal loading case (A) and a disparate loading case (E). The simulations were compared to in vitro measurements of fiber mechanics (blue circles) by Klotzsch et al. The tension in the equally loaded fiber (B) rose more quickly to a higher value than the disparate loading case (F). The difference between experiment and simulation was quantified by the ratio between force of the simulations (Fin silico) and force of the experiment (Fin vitro) for the equal loading case (C) and the disparate loading case (G). Stress was calculated for the simulation with the assumption of constant volume extension. Stress in the equally loaded fiber (D) was higher than the in vitro measurements over the strain range. Stress in the disparately loaded fiber (H) was lower than the equally loaded fiber and better approximated the in vitro data. (TIF) [file pcbi.1002845.s002.tif]
